# Research on the Mechanism and Prevention of Hypertension Caused by Apatinib Through the RhoA/ROCK Signaling Pathway in a Mouse Model of Gastric Cancer

**DOI:** 10.3389/fcvm.2022.873829

**Published:** 2022-06-23

**Authors:** Wenjuan Wang, Caie Li, Chenchen Zhuang, Haodong Zhang, Qiongying Wang, Xin Fan, Miaomiao Qi, Runmin Sun, Jing Yu

**Affiliations:** Department of Hypertension Center, Lanzhou University Second Hospital, Lanzhou, China

**Keywords:** hypertension, apatinib, gastric cancer, RhoA/ROCK signaling pathway, Y27632

## Abstract

Hypertension is one of the main adverse effects of antiangiogenic tumor drugs and thus limits their application. The mechanism of hypertension caused by tyrosine kinase inhibitors (TKIs) targeting vascular endothelial growth factors is mainly related to inhibition of the nitric oxide (NO) pathway and activation of the endothelin pathway, as well as vascular rarefaction and increased salt sensitivity; consequently, prevention and treatment differ for this type of hypertension compared with primary hypertension. Apatinib is a highly selective TKI approved in China for the treatment of advanced or metastatic gastric cancer. The RhoA/ROCK pathway is involved in the pathogenesis of hypertension and mediates smooth muscle contraction, eNOS inhibition, endothelial dysfunction and vascular remodeling. In this study, *in vivo* experiments were performed to explore whether the RhoA/ROCK signaling pathway is part of a possible mechanism of apatinib in the treatment of gastric cancer-induced hypertension and the impairment of vascular remodeling and left ventricular function. Y27632, a selective small inhibitor of both ROCK1 and ROCK2, was combined with apatinib, and its efficacy was evaluated, wherein it can reduce hypertension induced by apatinib treatment in gastric cancer mice and weaken the activation of the RhoA/ROCK pathway by apatinib and a high-salt diet (HSD). Furthermore, Y-27632 improved aortic remodeling, fibrosis, endothelial dysfunction, superior mesenteric artery endothelial injury, left ventricular dysfunction and cardiac fibrosis in mice by weakening the activation of the RhoA/ROCK pathway. The expression of RhoA/ROCK pathway-related proteins and relative mRNA levels in mice after apatinib intervention were analyzed by various methods, and blood pressure and cardiac function indexes were compared. Endothelial and cardiac function and collagen levels in the aorta were also measured to assess vascular and cardiac fibrosis and to provide a basis for the prevention and treatment of this type of hypertension.

## Introduction

Continued progress in cancer treatment technology has prolonged the survival of cancer patients, and cardiovascular disease has gradually emerged as an important cause of death in cancer patients. This phenomenon is partly due to the cardiovascular toxicity of antitumor therapy, which leads to cardiovascular problems in patients with cancer (1, 2). Hypertension is one of the most common adverse events caused by antiangiogenic antitumor drugs. Increased blood pressure (BP) not only causes discontinuation of antitumor therapy but also exacerbates the occurrence of cardiovascular events (3). A phase III clinical trial has proven that apatinib is safe and effective in patients with advanced gastric cancer (4).

Apatinib is a highly selective tyrosine kinase inhibitor (TKI) of vascular endothelial growth factor receptor-2 (VEGFR2) (5–7). Little is known about the mechanism of TKI-induced hypertension. The proposed mechanisms include activation of the endothelin (ET) system and inhibition of the nitric oxide (NO) pathway, vascular rarefaction, renal function impairment caused by glomerular injury, and increased salt sensitivity (8, 9). Endothelin (ET)-1 is a potent vasoconstrictor peptide produced and released primarily by endothelial cells (10) that inhibits endothelial nitric oxide synthase (eNOS) and, in turn, NO production (11, 12). Rho-kinase was first identified as a serine/threonine kinase that specifically binds to GTP-RhoA (an activated form of RhoA) (13).

The RhoA-associated kinase (RhoA/ROCK) signaling pathway plays an important role in the pathogenesis of salt-sensitive hypertension and hypertension-induced cardiac hypertrophy and enhances the vasoconstriction effect of ET-1. Moreover, activation of this pathway reduces endothelial NO production and vasodilation function, ultimately promoting the occurrence of hypertension (14–16). A high salt intake can significantly increase the elevation of BP induced by sunitinib (17). Therefore, it is speculated that the RhoA/ROCK pathway is involved in the occurrence of hypertension induced by TKIs and that ROCK pathway inhibitors may have therapeutic effects on this hypertension. However, no related research has been reported.

The RhoA/ROCK signaling pathway is deeply involved in arterial hypertension, cardiovascular–renal remodeling, hypertensive nephropathy and posttransplant hypertension (18). RhoA mainly interacts with two isoforms of Rho-associated coiled-coil domain-containing protein kinases (ROCK1 and ROCK2) to induce the phosphorylation and inhibition of myosin light chain phosphatase (MLCP) (19). ET-1, as an upstream effector, increases the phosphorylation level of MLC through the RhoA/ROCK pathway, enhances vascular endothelial oxidative stress, increases peroxide production, reduces NO production through this pathway, and enhances vasoconstriction, leading to hypertension (20–22). Moreover, RhoA/ROCK-mediated phosphorylation of MYPT-1 (p-MYPT-1) at specific residues is associated with inhibition of MLCP, leading to an increase in smooth muscle contraction (23–25).

The incidence of hypertension caused by apatinib is approximately 35.2%, of which approximately 4.5% is grade 3-4 hypertension (4, 26). However, the pathogenesis of this type of hypertension is not the same as that of essential hypertension, and the specific mechanism is unclear. Therefore, it is very important to explore the pathogenesis of apatinib-induced hypertension and to identify new specific therapeutic drugs that can promote the application of apatinib, prevent vascular events and improve patient prognosis. The goals of this study were to apply gastric cancer modeling technology to simulate the real *in vivo* environment to explore whether the RhoA/ROCK signaling pathway is involved in the occurrence of this type of hypertension and whether ROCK inhibitors have therapeutic effects.

## Materials and Methods

### Cell Lines and Culture

The human gastric adenocarcinoma cell line MKN-45 was purchased from and validated by American Type Culture Collection (ATCC; Manassas, VA, United States). Cells were cultured following instructions from ATCC in Roswell Park Memorial Institute medium (RPMI 1640, Invitrogen, Shanghai, China). All cells were supplemented with 10% fetal bovine serum (FBS, Invitrogen, Shanghai, China), 100 U/ml penicillin and 100 U/ml streptomycin (Invitrogen, Shanghai, China) at 37°C in a humidified environment of 5% CO_2_. All cells were used within 6 months after resuscitation and were free of mycoplasma.

### Animals

All experiments were approved by the Ethics Committee of Lanzhou University Second Hospital (license number: D2019-101), and all manipulations were performed in accordance with the Guide for the Care and Use of Laboratory Animals. Female BALB/C nude mice (4–6 weeks old, 20 ± 5 g) were provided by Beijing Vital River Laboratory Animal Technology Co., Ltd. (Beijing, China; SCXK2012-0001).

### Animal Experiments

The procedure used for establishing mouse orthotopic mammary xenograft tumors was described recently (27). Nude mice and MKN-45 were used for gastric cancer modeling.

Mice were fed in SPF-class barrier facilities that met the requirements of GB14925 (2010 Experimental Animal Environment and Facilities). All mice were reared on sterilized food and water and were housed eight mice per cage in standard polycarbonate cages with controlled temperature and humidity and a 12-h light and dark cycle (28). Adaptive feeding was performed for 1 week before the experiment, and all efforts were made to minimize suffering.

### Nude Mouse Xenograft Tumor Assays

Mice were randomly placed into normal (NR) (*n* = 6 mice) or tumour-bearing (TB) groups. MKN-45 cells (1 × 10^7^ in 100 μL of medium) and 50% matrix glue were inoculated subcutaneously into the right front armpit of the mice. Mice in the intervention groups were given apatinib 50 mg/kg/day. When the tumor volumes reached approximately 100–200 mm^3^, the TB group was randomly divided into four groups, with each group (*n* = 6 mice) having similar average tumor volumes: (i) control, 2 ml of normal saline gavage; (ii) high-salt diet (a custom 8% high-salt feed);

(iii) high-salt diet (a custom 8% high-salt feed) + 10 mg/kg Y27632 administered *via* intraperitoneal injection; (iv) high-salt diet (a custom 8% high-salt feed) + 50 mg/kg apatinib dissolved in 2 ml of normal saline by gavage; (v) 50 mg/kg apatinib dissolved in 2 ml of normal saline by gavage; and (vi) 50 mg/kg apatinib dissolved in 2 ml of normal saline by gavage + 10 mg/kg Y27632 administered *via* intraperitoneal injection. The mice were anesthetized by inhalation of 1.0–2.5% isoflurane on day 28 after drug intervention to collect their blood, and tissues were immediately frozen in liquid nitrogen. The anesthetized mice were sacrificed by carbon dioxide asphyxiation. The tumors were isolated and weighed. The tumor-free body weight at the last time point was calculated using the following formula: tumor-free body weight = (body weight with tumor)–(tumor weight) (29).

### Blood Pressure Analysis

The tail pressure of the mice was measured every 3 days and before sacrifice. An IITCMRBP tail cuff blood pressure system (Yuyan Instruments, Shanghai, China) was used for measurements; the mice were placed within the instrument while the temperature was set to 36°C. After 15–20 min of relative quiet, the systolic blood pressure (SBP), diastolic blood pressure (DBP) and mean blood pressure (MBP) were collected when the mice were awake. Measurements were performed three times every 15 s, and the average value was recorded.

### Echocardiography and Heart Weight Analysis

Mouse cardiac function was measured using a high-resolution echocardiography imaging system (Vinno 6 Lab, Vinno Technology, Beijing, China) following a sedation protocol (inhaled isoflurane 2.5%). Two trained, blinded operators (W.J.W. and Q.Y.W.) performed echocardiography to evaluate cardiac function. The ejection fraction (EF) and fractional shortening (FS) were calculated and considered systolic function. The left ventricular internal dimension-diastole (LVIDd) and left ventricular internal dimension-systole (LVIDs) were measured for left ventricular dilation. The mice were weighed throughout the experiment to properly administer the correct treatment dose. Heart weight (HW), tibia length (TL) and left ventricular mass (LVM) were measured, including the septum. Then, the heart weight-to-tibia length ratio (HW/TL) and left ventricular mass to-tibia length ratio (LVM/TL) were calculated to evaluate cardiac hypertrophy *in vivo*.

### Histopathology and Immunohistochemistry

Tissues were fixed in 4% paraformaldehyde in PBS at room temperature for 24–48 h, embedded in paraffin (Solarbio, Beijing, China), sectioned into 3- to 5-μm pieces and placed in neutral resin to seal the film. Haematoxylin–eosin (HE) staining was performed to observe cell structure and organizational hierarchy. The experimental steps were carried out in strict accordance with the kit instructions (Solarbio, Beijing, China).

Masson’s trichrome staining was used to evaluate the area of fibrosis. Staining was performed on each heart and aorta section according to the standard procedure of the Masson’s trichrome staining kit (Solarbio, Beijing, China). Immunohistochemistry was performed by applying the UltraSensitive™ SP (mouse/rabbit) IHC KIT-9710 protocol method (Manxin, Fuzhou, China) with anti-COL I (Proteintech, Wuhan, China) and anti-COL III (Proteintech, Wuhan, China) antibodies to each aorta section. Images were collected using a Nikon Eclipse 80i (Nikon, Japan), and subsequent analysis was performed using ImageJ software.

### Western Blotting

Western blotting was performed as previously described (29). Total protein was extracted in radioimmunoprecipitation assay (RIPA) buffer and phenylmethanesulfonyl fluoride (PMSF) (Beyotime Biotechnology, Shanghai, China) at a ratio of 100:1. The protein concentration was measured by a BCA assay kit (Biosharp, AnHui, China), and the protein was loaded onto a 5 × sodium dodecyl sulfate–polyacrylamide gel electrophoresis (SDS–PAGE) gel; a loading buffer (Biosharp, Anhui, China) at a volumetric ratio of 5:1 was used.

After denaturation and SDS–PAGE electrophoresis, the proteins were transferred to polyvinylidene fluoride (PVDF) membranes, which were blocked with 5% non-fat dried milk (Sigma, Shanghai, China) and incubated with primary antibodies including anti-ROCK1, anti-RhoA, anti-ROCK2, anti-α-SMA, anti-eNOS, anti-iNOS anti-ET-1, anti-CD31 phospho-MYPT-1, MYPT-1 (Abcam, Shanghai, China), phospho-MLC, MLC (Cell Signaling Technology), and GAPDH (Proteintech, WuHan, China) overnight at −20°C. The PVDF membranes were washed with TBST and then with secondary antibodies. Finally, the membranes were washed again and incubated in chemiluminescent ECL substrate (Fisher). The images were analyzed by ImageJ software, and the results were normalized to those of GAPDH. Analysis was performed using ImageJ software.

### qRT–PCR Analysis

Total RNA was extracted using TRIzol (Takara, Japan). For mRNA quantification, a PrimeScript™ RT reagent Kit (Takara) was used to prepare cDNA. Real-time PCR was performed with SYBR Green (Bio-Rad, United States) on a Bio-Rad CFX-96 real-time system (Bio-Rad, Hercules, California, United States). Relative RNA expression was determined by the relative standard curve method (2^–Δ^ ^Δ^ *^CT^*). PCR primers were synthesized by Sangon Biotech (Shanghai, China). The following primers were used in this study: RhoA (forward: 5′- GAG TTG GAC TAG GCA AGA AAC TC-3′; reverse: 5′-ACC CAA ACC CTC ACT GTC TTC -3′); ROCK1 (forward: 5′-TGG GAG AGT GAG CCT GTT CT -3′; reverse: 5′-TAG AGG TGG TCT AGC CCT GCA T -3′); ROCK2 (forward: 5′-TGT TAG GGT TCC CAG GGT GA -3′; reverse: 5′-AGA AGC TCG GAA GCT ACG TG -3′); eNOS (forward: 5′-TCT GCG ATG TCA CTA TG -3′; reverse: 5′-ATG ACG TCA CCG GCT TCA TC -3′); iNOS (forward: 5′- TCT AGT GAA GCA AAG CCC AAC -3′; reverse: 5′- GGA ACA TTC TGT GCT GTC CCA -3′); ET-1 (forward: 5′-CTA CGA AGG TTG GAG GCC AT -3′; reverse: 5′-TCG GTT GTG CGT CAA CTT CT -3′); COLI (forward: 5′- TCG GAC TAG ACA TTG GCC CT -3′; reverse: 5′- TCG TCT GTT AGG GTT GG -3′); COLIII (forward: 5′- CCA AGG CTG CAA GAT GGA TG-3′; reverse: 5′- TGT CCA GTG CTT ACT TG-3′); and CX43 (forward: 5′- TCT GAG AGC CCG AAC TCT CCT T -3′; reverse: 5′-CTG GGC ACC TCT CTT TCA CTT –3′).

### Statistical Analysis

All experiments were performed at least three times. SPSS version 23 (IBM, Armonk, New York, United States) and GraphPad Prism version 8.0 (San Diego, CA, United States) were used for statistical analysis. Data are presented as the mean ± standard error of the mean (SEM) or geometric means with 95% confidence intervals (CIs). Differences between two independent groups were determined using an unpaired Student’s *t*-test, while one-way or two-way ANOVA followed by either Dunnett’s or Tukey’s *post-hoc* test was used to compare means across multiple groups. A *p*-value of less than 0.05 was considered statistically significant.

## Results

### Apatinib Treatment Increases Mouse Weight and Decreases Tumor Volume, While Y27632 Treatment Does Not Affect Body Weight or Tumor Volume

**FIGURE 1:**
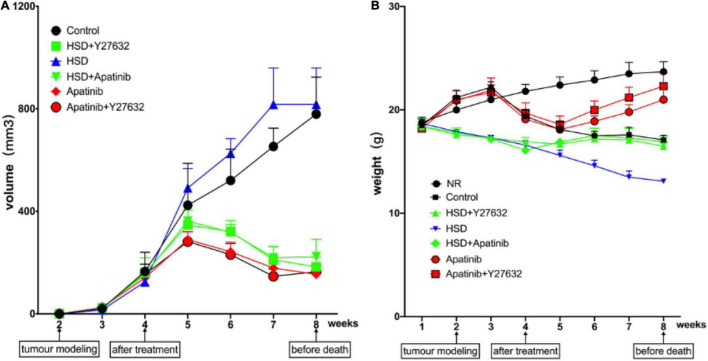
Changes in body weight and tumor volume in mice. **(A)** The trend of tumor volume after modeling (mm^3^). Tumor volume showed a decreasing trend in the fifth week after apatinib intervention for 1 week and showed a decreasing trend after Y27632 treatment. However, there was no statistical significance for the influence of the two drugs on tumor volume (*P* > 0.05). Similar trends were observed when apatinib and Y27632 were given with a high-salt diet. **(B)** Change trend of body weight at baseline, after modeling, after drug intervention and at death (g). Body weight showed an increasing trend in the sixth week after apatinib intervention for 2 weeks and showed an increasing trend after Y27632 treatment. However, there was no statistical significance in the influence of the two drugs on body weight (*P* > 0.05). Similar trends were observed when apatinib and Y27632 were given with a high-salt diet. Data are representative of three independent measurements. Mean ± SEM.

Weight changes in each group, including weight at baseline, after tumor modeling, after treatment, and before death, were recorded weekly (Figure 1A).

Over the course of the experiment, body weight increased gradually in the NR group and reached 23.7 ± 1 g, while body weight in the control group began to show a downward trend after tumor modeling (Figure 1A) and reached 17.1 ± 0.5 g before death. Body weight in the HSD group tended to decrease continually and reached 13.7 ± 0.21 g before death, while body weight in the HSD + Y27632 and HSD + apatinib groups increased for 1 week after treatment and reached 16.5 ± 0.8 g before death. However, body weight in the apatinib group and the apatinib + Y27632 group tended to decrease after tumor modeling but gradually increased in the week after treatment (Figure 1A). Compared with that in the NR group, body weight tended to decrease in the control group (*p* < 0.001). Compared with that in the control group, body weight decreased in the HSD group (*p* < 0.001) but increased slightly in the HSD + Y27632, HSD + apatinib, and apatinib + Y27632 groups (*p* < 0.001). There was no significant difference in weight between the apatinib and apatinib + Y27632 groups or between the HSD + apatinib and HSD + Y27632 groups.

The changes in tumor volume in each group were evaluated and recorded after tumor modeling. The tumor volumes in the control and HSD groups increased over time and reached 779.4 ± 144.1 mm^3^ by death, while those in the HSD + apatinib and HSD + Y27632 groups and the apatinib and apatinib + Y27632 groups decreased in the week after treatment (Figure 1B). Compared with the control group, tumor volume decreased in the apatinib and apatinib + Y27632 groups (*p* < 0.001), but tumor volume did not differ significantly between the apatinib and apatinib + Y27632 groups (Figure 1B). Compared with that in the HSD group, tumor volume decreased in the HSD + apatinib and HSD + Y27632 groups (*p* < 0.001), but tumor volume did not differ significantly between the latter two groups (Figure 1B).

### Apatinib Induces Elevated Blood Pressure and Can Enhance High-Salt Diet-Induced Elevated Blood Pressure, and Y27632 Mitigates These Effects

**FIGURE 2:**
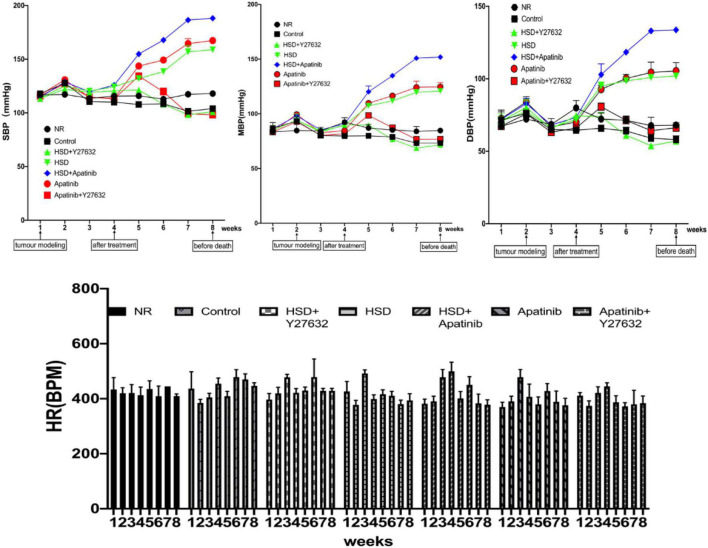
Changes in the BP of mice in each group. After 1 week of gastric cancer modeling, BP showed a temporary upward trend and returned to the baseline BP level in the third week. Apatinib and the high-salt diet increased BP from the fourth to the eighth week of treatment, and the increase in BP after the high-salt diet combined with apatinib treatment was most obvious and reached a plateau between weeks 7 and 8. Y27632 mitigated the apatinib-induced elevation in BP in the apatinib (*P* < 0.001) and high-salt diet (*P* < 0.001) groups. Data are representative of three independent measurements. Mean ± SEM. BP, blood pressure; HR, heart rate; SBP, systolic blood pressure; DBP, diastolic blood pressure; MBP, mean blood pressure.

BP was recorded weekly in each group. The baseline BP was 114.50/67.17 ± 1.78/6.51 mmHg, and the baseline MBP was 83.50 ± 4.60 mmHg in all groups. After tumor modeling, there was a slight increase in BP in all groups, which gradually decreased to baseline at week 3. There were no significant differences in SBP, DBP or MBP in the control group compared with the NR group, while BP significantly increased in the apatinib group from the fifth week and the HSD and HSD + apatinib groups from the third week, regardless of whether it was SBP, DBP or MBP, reaching a plateau after 7 weeks (*p* < 0.001). The increase in BP was most significant in the HSD + apatinib group (*p* < 0.001) (Figure 2). Before the mice were killed, SBP/DBP was 158.83/101.83 ± 2.71/1.54 mmHg, 188.17/133.67 ± 2.07/1.17 mmHg, and 167.33/105.33 ± 2.11/5.78 mmHg in the HSD, HSD + apatinib and apatinib groups, respectively. Notably, Y27632 significantly mitigated the apatinib-induced and HSD-induced elevation in BP. SBP/DBP was 101/57 ± 1.67/1.84 mmHg in the HSD + Y27632 group, and the BP was lower than that in the HSD group (*p* < 0.001). The BP of the apatinib + Y27632 group was 98.17/66.17 ± 1.25/3.51 mmHg, which was lower than that of the apatinib group (*p* < 0.001).

### Apatinib Activates the RhoA/ROCK Signaling Pathway in Mouse Models of Gastric Cancer

**FIGURE 3:**
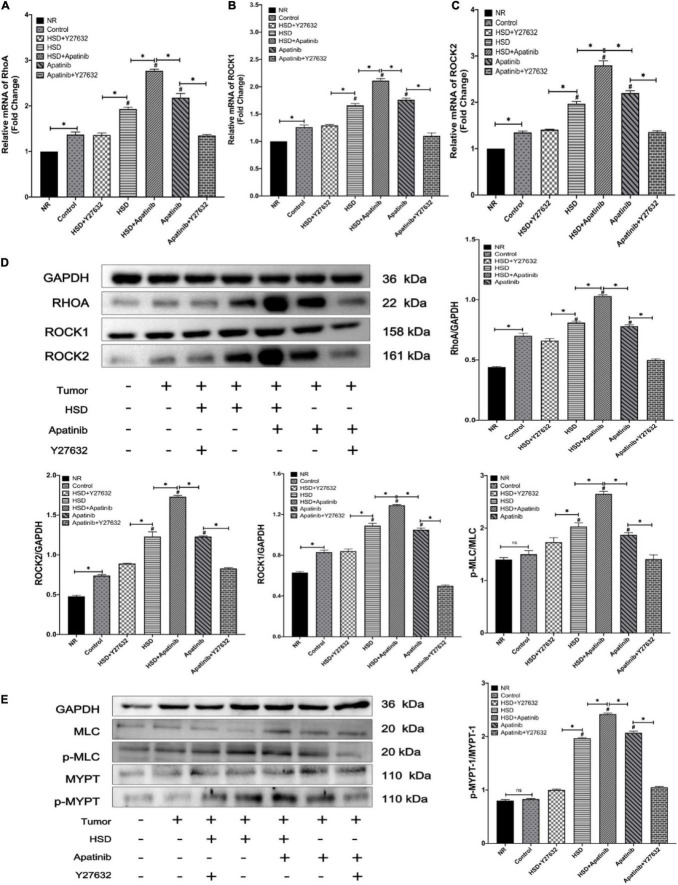
Expression of the RhoA/ROCK pathway was detected by qRT–PCR and western blotting. **(A–C)** The levels of RhoA and Rho-associated coiled-coil domain-containing protein kinase mRNAs. **(E)** The protein expression levels of RhoA/Rho-associated coiled-coil domain-containing protein kinase signaling pathway members. **(E)** Changes in the phosphorylation levels of MLC and MYPT1 after apatinib and high-salt diet intervention. Apatinib and HSD upregulated the expression of RhoA ROCK1 and ROCK2 proteins and mRNAs. Administration of Y27632 reduced the phosphorylation levels of MLC and MYTP downstream of the RhoA/ROCK signaling pathway induced by apatinib and a high-salt diet (*n* = 6 in each group). Data are representative of three independent measurements. Mean ± SEM. ^∗^*P* less than 0.001. **^#^***P* less than 0.001, comparison between each group and the control group. ROCK, Rho-associated coiled-coil domain-containing protein kinase. MLC, myosin light chain phosphatase. MYPT, myosin phosphatase target subunit.

RhoA/ROCK signaling pathway-related mRNA levels and protein expression were detected and measured by western blotting and qRT–PCR. The relative mRNA levels and protein expression of RhoA, ROCK1, and ROCK2 in mouse aortas increased after HSD, and this effect of HSD was enhanced by apatinib (*p* < 0.001) (Figures 3A–D. Y27632 treatment decreased the mRNA and protein expression levels of RhoA, ROCK1 and ROCK2 in mouse aortas compared with apatinib treatment alone (*p* < 0.001). The same effect was observed in the HSD + Y27632 group (*p* < 0.001). This effect was more obvious for ROCK2 than for the mRNA and protein levels of ROCK1, and ROCK1 expression was still higher than that in the control group (Figures 3A–D).

Subsequently, the phosphorylation of MLC and MYPT-1 in this pathway was assessed. p-MLC/P-MLC/and P-MYPT/MYPT-1 protein levels were increased in the aortas of mice treated with apatinib, and the HSD + apatinib group showed a more obvious trend (*p* < 0.001) (Figure 3E). However, this effect was mitigated when Y27632 was administered. Similarly, administration of Y27632 after HSD reduced the protein expression of p-MLC and P-MYPT-1 (*p* < 0.001) (Figure 3E). That is, apatinib induced the activation of the RhoA/ROCK pathway by stimulating the phosphorylation of MLC and MYPT-1, while treatment with Y27632 inhibited the expression of phosphorylated proteins.

### Apatinib Upregulates ET-1 and Downregulates the NO System in Mouse Models of Gastric Cancer

**FIGURE 4:**
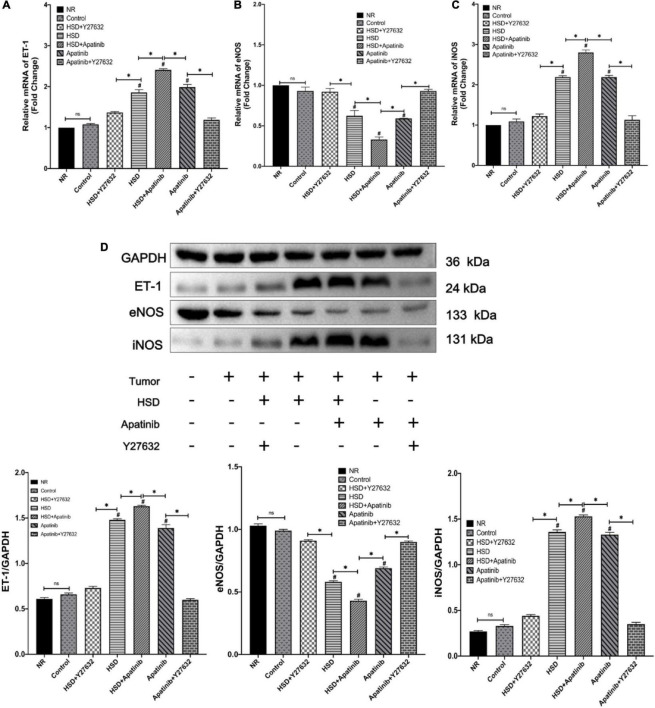
Expression of proteins related to vascular contraction and vasodilation systems was detected by qRT–PCR and western blotting. **(A–C)** The levels of vascular contraction and vasodilation mRNAs. **(D)** The protein expression levels of vascular contraction and vasodilation systems. The protein levels and mRNAs of ET-1 were upregulated with the administration of apatinib and a high-salt diet. Y27632 mitigated both. The protein levels and mRNAs of nitric oxide synthase showing that apatinib and a high-salt diet downregulated eNOS and upregulated iNOS, while the effect was mitigated by Y27632 (*n* = 6 in each group). Data are representative of three independent measurements. Mean ± SEM. ^∗^*P* less than 0.001. ^#^*P* less than 0.001, comparison between each group and the control group. ET-1, endothelin-1, eNOS, endothelial nitric oxide synthase; iNOS, inducible nitric oxide synthase.

ET-1 and NO levels in mouse aortas were assessed by qRT–PCR and western blotting (Figures 4A–D). The mRNA and protein expression of ET-1 increased after HSD + apatinib treatment and was higher than the levels in the apatinib and HSD groups (*p* < 0.001). Moreover, Y27632 attenuated the increase in ET-1 induced by apatinib, and ET-1 levels were lower in the HSD + Y27632 group than in the HSD group (*p* < 0.001) (Figures 4A–D). The mRNA and protein expression of iNOS increased in the HSD + apatinib group compared with the apatinib and HSD groups (*p* < 0.001) and decreased after Y27632 treatment (*p* < 0.001) (Figures 4A–D). In contrast, eNOS protein expression and relative mRNA levels in mouse aortas decreased after apatinib treatment but were higher than those in the aortas of mice treated with HSD + apatinib (*p* < 0.001). However, after Y27632 treatment, eNOS protein expression and relative mRNA levels were increased compared with apatinib (*p* < 0.001) and were also higher in the HSD + Y27632 group than in the HSD group (*p* < 0.001) (Figures 4A–D).

### Apatinib Induces Vascular Remodeling in Mouse Models of Gastric Cancer, and Y27632 Ameliorates This Effect

**FIGURE 5:**
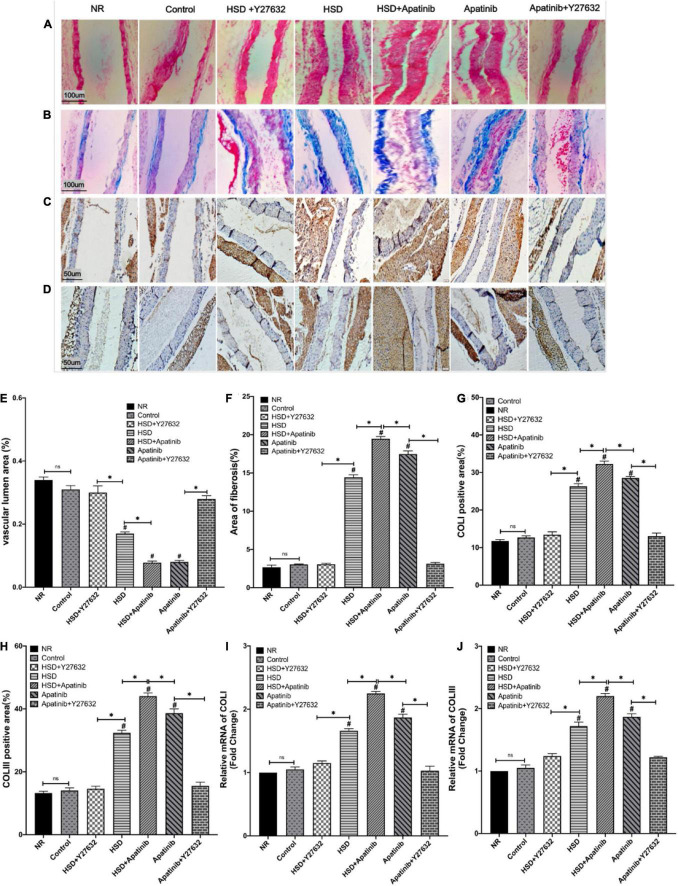
Histological sections of aortic fibrosis *in vivo*. **(A)** Sections stained with H&E. **(B)** Sections stained with Masson staining. **(C,D)** COLI and COLIII immunohistochemical staining. **(E)** The subluminal area of the vessels (%) was decreased in the HSD, HSD + apatinib, and apatinib groups. However, after Y27632 treatment, the area of the vessels was increased. **(F)** The area of Masson staining for fibrosis (%) was increased in the HSD, HSD + apatinib and apatinib groups; after Y27632 treatment, the area of the vessels was decreased. **(G,H)** Positive signal areas of COLI and COLIII (%) were increased in the HSD, HSD + apatinib and apatinib groups. After Y27632 treatment, the vessel diameter was decreased, which is consistent with the fibrosis biomarkers, including COLI and COLIII, detected at the mRNA level **(I,J)** (*n* = 6 in each group). Data are representative of three independent measurements. Mean ± SEM.^∗^*P* less than 0.001. ^#^*P* less than 0.001, comparison between each group and the control group. COLI, collagen I; COLIII, collagen III.

Haematoxylin–eosin staining showed that the aortic lumen was narrowed and the vascular wall was thickened in mice treated with HSD and apatinib, and these effects were enhanced in the HSD + apatinib group (*p* < 0.001). The lumen area was increased in mice treated with Y27632, and the wall was thinner than that in mice treated with apatinib (*p* < 0.001) (Figures 5A,E). The vascular wall was also thinner in the HSD + Y27632 group than in the HSD group (*p* < 0.001) (Figures 5A,E). Masson staining showed that the degree of aortic blue staining increased after HSD and apatinib treatment, and this effect was enhanced in the HSD + apatinib group (*p* < 0.001). The area of blue staining decreased after Y27632 treatment compared with apatinib treatment (*p* < 0.001) and was also decreased in the HSD + Y27632 group (*p* < 0.001) (Figures 5B,F). The observed patterns of collagen overexpression inspired us to investigate the mechanical metrics of the mid-aorta. Immunohistochemistry showed that the positive signals of COLI and COLIII increased significantly after apatinib treatment and HSD, and the HSD + apatinib group showed the highest positive signal (*p* < 0.001). After treatment with Y27632, the positive signal was lower, and the fibrosis was relieved compared with apatinib treatment (*p* < 0.001). Fibrosis was also lower in the HSD + Y27632 group than in the HSD group (*p* < 0.001) (Figures 5C,D,G,H). From the mRNA levels, the results were similar to those of immunohistochemistry (*p* < 0.001) (Figures 5I,J).

### Apatinib Reduced the Vascular Density of the Superior Mesenteric Artery, and This Effect Was Attenuated by Y27632 in Mouse Models of Gastric Cancer

**FIGURE 6:**
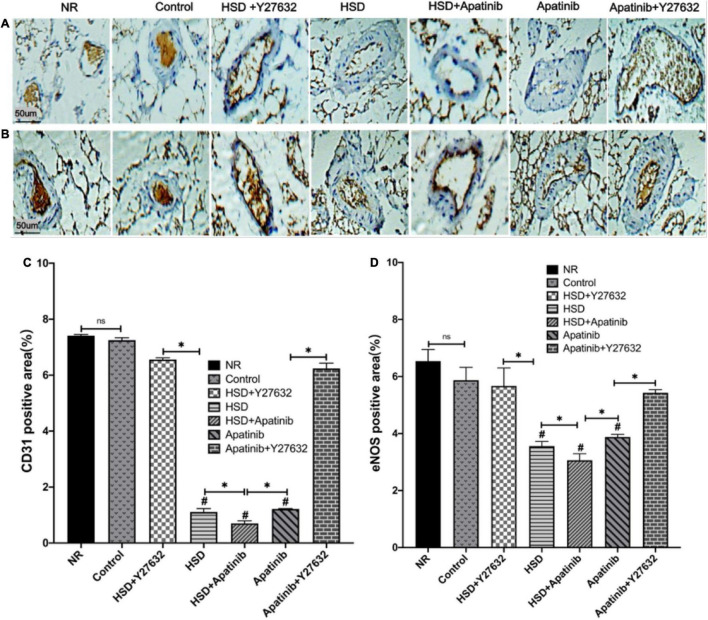
Histological sections of the superior mesenteric artery *in vivo*. **(A,B)** CD31 and eNOS immunohistochemical staining. **(C,D)** Positive signal areas of CD31 and eNOS assessed by immunohistochemistry (%). Positive signal areas of CD31 and eNOS (%) were decreased in the HSD, HSD + apatinib and apatinib groups, and after Y27632 treatment, the area of the vessels was increased (*n* = 6 in each group). Data are representative of three independent measurements. Mean ± SEM. ^∗^*P* less than 0.001. ^#^*P* less than 0.001, comparison between each group and the control group. eNOS, endothelial nitric oxide synthase. CD31, Platelet endothelial cell adhesion molecule-1 (PECAM-1).

To determine whether the vascular remodeling in the superior mesenteric artery of mice was due to a reduction in vascular density, we performed CD31 staining. The results of immunohistochemistry showed that the positive signals of CD31 and eNOS decreased significantly after HSD and apatinib treatment, and the downward trend of the positive signal in the HSD + apatinib group was the most obvious (*p* < 0.001). The positive signals of CD31 and eNOS were stronger in the apatinib + Y27632 group than in the apatinib group (*p* < 0.001) (Figures 6A–D) but lower in the HSD + Y27632 group than in the HSD group (*p* < 0.001). The stronger positive signals of CD31 and eNOS in the apatinib + Y27632 and HSD + Y27632 groups suggest that administration of Y27632 can improve vascular injury to the superior mesenteric artery (Figures 6A–D).

### Y27632 Improves Hypertension-Induced Cardiac Hypertrophy Caused by Apatinib in Mouse Models of Gastric Cancer

**FIGURE 7:**
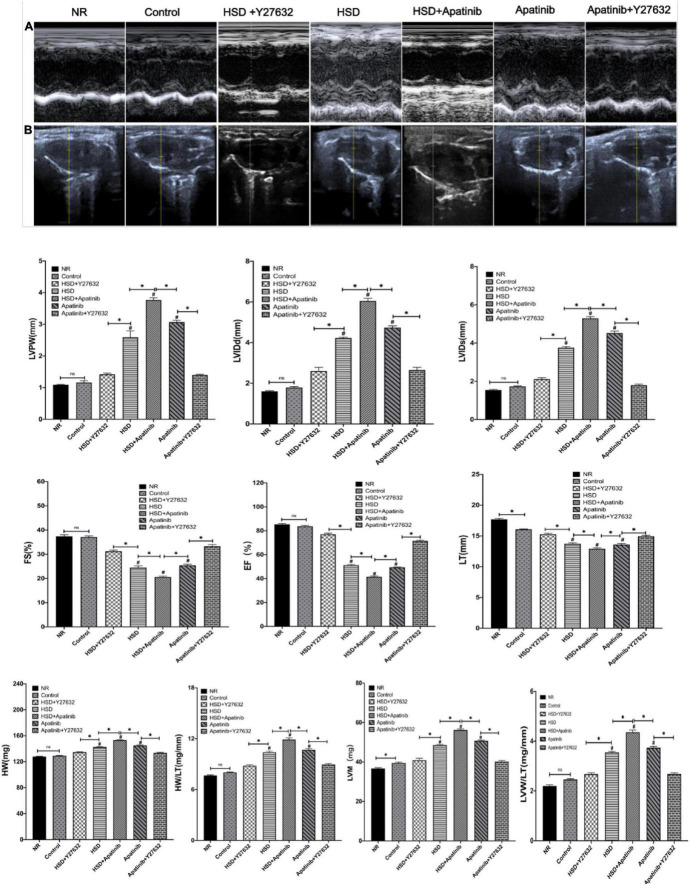
The effects of apatinib and Y27632 on left ventricular function *in vivo*. **(A)** M-mode echocardiography of the left ventricular chamber, including the FS (%), EF (%), lVPW, LVIDd (mm) and LVIDs (mm). **(B)** Long axis section of the parasternal left ventricle. In the HSD, HSD + apatinib and apatinib groups, LVPW, LVIDd, LVIDs, HW (mg), and LVM (mg) increased, while after Y27632 treatment, these indicators were decreased. The FS and EF have the opposite trends. Then, the ratios of HW/TL and LVM/TL were calculated to evaluate cardiac hypertrophy, which increased in the HSD, HSD + apatinib and apatinib groups, and after Y27632 treatment, the ratios were decreased (*n* = 6 in each group). Data are representative of three independent measurements. Mean ± SEM. ^∗^*P* less than 0.001. ^#^*P* less than 0.001, comparison between each group and the control group. LVPW, left ventricular posterior wall thickness; LVIDd, left ventricular internal dimension-diastole; LVIDs, left ventricular internal dimension-systole; FS, fraction shortening; EF, ejection fraction; HW, heart weight; LVM, left ventricular mass; TL, tibial length.

Left ventricular function in each experimental group was evaluated using the long-axis view of the mouse sternum and M-mode echocardiography (Figures 7A,B). There were no significant differences in functional indexes between the NR and control groups, suggesting that tumor modeling may not cause damage to cardiac function in the short term. In the HSD and apatinib groups, LVPW, LVIDd, LVIDs, HM, and LVM, which were measured for left ventricular dilation, increased; these elevations were most pronounced in the HSD + apatinib group (*p* < 0.001) (Figure 7). FS and EF decreased, indicating left ventricular systolic dysfunction, and these decreases were significant in the HSD + apatinib group compared with the HSD and apatinib groups (*p* < 0.001) (Figure 7). The ratio of HW/TL and LVW/TL used to evaluate cardiac hypertrophy increased in the apatinib group and the HSD group (*p* < 0.001), and the increase was most significant in the HSD + apatinib group (*p* < 0.001). After treatment with Y27632, LVPW, LVIDd, LVIDs, HW, LVM and the ratios of HW/TL and LVW/TL decreased (*p* < 0.001), and EF and FS increased (*p* < 0.001) compared with those in the apatinib and HSD groups, which suggests that Y27632 can improve left ventricular dysfunction induced by apatinib and HSD (Figure 7).

### Y27632 Improves Cardiac Fibrosis Caused by Apatinib in Mouse Models of Gastric Cancer

**FIGURE 8:**
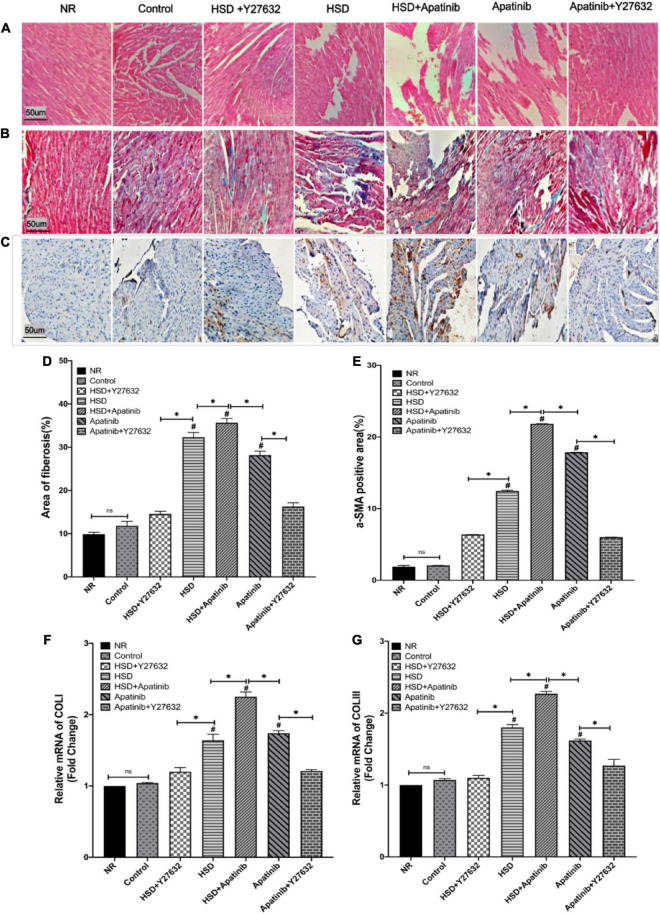
Histological sections of myocardial fibrosis *in vivo*. **(A)** Sections stained with H&E. **(B)** Sections stained with Masson staining. **(C)** α-SMA immunohistochemical staining. **(D)** The area of Masson staining for fibrosis (%) was increased in the HSD, HSD + apatinib and apatinib groups. After Y27632 treatment, the area of fibrosis was decreased. **(E)** Positive signal areas of α-SMA (%) have the same result as Masson staining. **(F,G)** Fibrosis indicators, including COLI and COLIII, were detected at the mRNA level, which was consistent with the Masson staining and α-SMA immunohistochemical staining (*n* = 6 in each group). Data are representative of three independent measurements. Mean ± SEM. ^∗^*P* less than 0.001. ^#^*P* less than 0.001, comparison between each group and the control group. COLI, collagen I; COLIII, collagen III; α-SMA, alpha-smooth muscle actin.

HE staining showed that after treatment with HSD and apatinib, the myocardial fibers were disordered, and the gap increased (*p* < 0.001). These changes were most significant in the HSD + apatinib group compared with the HSD and apatinib groups (*p* < 0.001). After treatment with Y27632, the myocardial fibers were orderly, and the gap size was similar to those of the NR group and control group (Figure 8A).

Masson staining showed that the degree of cardiac tissue blue staining increased most significantly in the HSD + apatinib group compared with the HSD and apatinib treatment groups (*p* < 0.001), while the area of blue staining decreased in the apatinib + Y27632 and HSD + Y27632 groups, suggesting that cardiac fibrosis induced by apatinib and HSD was decreased (*p* < 0.001) (Figures 8B,D).

Positive signals for α-SMA in the immunohistochemical assay and the relative mRNA content of COLI and COLIII increased significantly after HSD and apatinib treatment, and these effects were enhanced in the HSD + apatinib group (*p* < 0.001). After treatment with Y27632, the positive signal was lower, and the mRNA level decreased in the apatinib + Y27632 and HSD + Y27632 groups, indicating that fibrosis induced by apatinib and HSD was relieved (Figures 8C,E–G).

## Discussion

Hypertension is one of the major adverse reactions in the treatment of gastric cancer with apatinib, which limits its application and may affect patient prognosis. A previous study showed that grade ≥3 treatment-related adverse events (TRAEs) were reported in 147 (77.4%) of 190 patients treated with apatinib, with the most common being hypertension (34.2%) (30). However, the pathogenesis of this type of hypertension is different from that of essential hypertension; the specific mechanism is not clear at present, and there is no specific drug for prevention and treatment. Therefore, we conducted *in vivo* experiments to explore the mechanism by which apatinib causes hypertension.

Previous studies have explored apatinib doses of 30–150 mg/kg per day in mice (31–35) and Y27632 doses of 10–30 mg/kg (36–38). Therefore, we used apatinib 50 mg/kg per day to explore the effects of apatinib on BP over a 28-day period and Y27632 10 mg/kg per day to observe the effect on apatinib-induced hypertension. These drug doses are much closer to those used in clinical settings.

In our *in vivo* experiments, we first confirmed that tumor volume was reduced after apatinib treatment, confirming previous studies of the obvious therapeutic effect of apatinib on gastric cancer in mice (39–42). Another problem with ROCK inhibitors is their impact on cancer therapy. ROCK blockade promotes cancer cell phagocytosis and induces antitumor immunity by enhancing T-cell priming *via* dendritic cells, thereby suppressing tumor growth in syngeneic tumor models (43). The ROCK pathway inhibitors include Y27632, fasudil (44), GSK429286 (45) and RKI1447 (46).

One of the most widely investigated ROCK inhibitors is Y-27632 (47), which is a selective small inhibitor of both ROCK1 and ROCK2 that suppresses the invasiveness and metastasis of rat and human hepatoma cells, bladder cancer cells, colorectal cancer cells, lung cancer cells, and cancer cell types (48–53). Accumulating evidence strongly suggests that ROCK is dominantly involved in the regulation of vascular contraction, and Y27632 most likely targets ROCK and normalizes contractile dysfunction (54). Therefore, Y27632 may counteract the BP-increasing effects of VEGF antagonists and promote their antineoplastic effects (3). We confirmed that the tumor volume decreased after Y27632 intervention, which means that Y27632 does not affect the antitumor efficacy of apatinib in the treatment of gastric cancer and is the best choice for the treatment of TKI-induced hypertension.

Increased ARHGEF11 expression in the Dahl salt-sensitive (SS) rats leads to actin cytoskeleton-mediated changes in cell morphology and cell function that promote hypertension and a decline in kidney function (15). As ARHGEF11 is known to act through RhoA, it was expected to be involved in the canonical (RhoA-ROCK) pathway (15). In Dahl SS rats, salt influences blood pressure, proteinuria, and renal hemodynamics through the Rho-ROCK pathway (55, 56). Previous studies have shown that TKI-induced hypertension and salt-sensitive hypertension have similar effects under high salt levels (14, 17, 57). On the other hand, Lankhorst et al. (58) demonstrated that sunitinib-induced hypertension has a similar mechanism to salt-sensitive hypertension and could be aggravated by a high salt intake. Therefore, we hypothesized that the RhoA/ROCK pathway may be involved in the occurrence of hypertension induced by TKIs and that ROCK pathway inhibitors may have a therapeutic effect on this hypertension. Therefore, mice fed an HSD were included in this experiment to explore the activation of the RhoA/ROCK signaling pathway by apatinib. The HSD activated the RhoA/ROCK signaling pathway, leading to increased BP. Moreover, the increase in BP was significantly greater in mice fed the HSD and treated with apatinib, confirming our hypothesis that apatinib can also affect the elevation of BP by activating the RhoA/ROCK signaling pathway. Additionally, after Y27632 was administered, BP decreased in the HSD + apatinib group, which means that the ROCK inhibitor Y27632 may be a potential therapeutic target for salt sensitivity-induced hypertension. Finally, there was no significant difference in heart rate between the groups (*p* > 0.05) (Figure 2), indicating that apatinib and the HSD had no significant effect on heart rate in mice with gastric cancer.

ROCK, a serine/threonine kinase and an important downstream effector of the small G protein RhoA, is an important factor in the development of smooth muscle tone (59). We previously demonstrated that apatinib increases BP in a rat model by activating the RhoA/ROCK signaling pathway and that this increase is reversed by Y27632 (3). On this basis, we carried out experiment to further simulate the real tumor environment. MKN-45 cells were used to build a tumor model. The tumor activated the RhoA/ROCK signaling pathway but not downstream phosphorylation, ET or the NO system. Only apatinib treatment caused further changes in the signaling pathway. This means that apatinib enhances the activation of the RhoA/ROCK signaling pathway in the tumor environment, causing hypertension. Chemokines and cytokines regulate the activation of RhoA to initiate the cytoskeletal changes required for the physical movement of the cells into the tumor stroma (60, 61). ROCK activity increases due to extracellular matrix rigidity in the tumor microenvironment and promotes a malignant phenotype *via* actomyosin contractility. The mechanical rigidity of the tumor microenvironment may drive ROCK signaling through distinct pathways to enhance the invasive migration required for cancer progression and metastasis (62). In this experiment, the tumor environment only caused increases in RhoA, ROCK1 and ROCK2, and other components of this signaling pathway were not elevated. However, after apatinib and HSD intervention, there was a superimposed elevation of these proteins that caused changes in other factors of the signaling pathway. The inactive form of RhoA (RhoA.GDP) is present in the cytosol bound to a guanine dissociation inhibitor (GDI). The activation of RhoA, such as *via* apatinib induction, is mediated by various Rho-specific guanine nucleotide exchange factors (RhoGEFs), which in turn promote the exchange of GDP for GTP. Upon GTP binding and activation, RhoA interacts with and stimulates the activity of downstream effectors, including ROCK. Active RhoA (GTP-bound) is in turn inactivated by GTPase-activating proteins (GAPs), which hydrolyze GTP to GDP (63). Y27632 is a structural analog of ATP, which has been shown to have potent inhibitory activity against both ROCK1 and ROCK2 (64). In this experiment, we further found that Y27632 inhibited ROCK and RhoA activity caused by activation of the RhoA/ROCK pathway induced by apatinib and HSD, which may be a potential therapeutic target for the treatment of hypertension caused by apatinib and HSD.

The results of qRT–PCR and western blot analyses revealed that apatinib treatment mediated the significant upregulation of RhoA, ROCK1 and ROCK2 expression. Activation of the RhoA/ROCK pathway maintains the level of MLC20 phosphorylation, an essential step in smooth muscle contraction, *via* the inhibition of MLC phosphatase activity by phosphorylation of the MLC phosphatase target subunit (MYPT-1) (59). Activating ROCK2 inhibits MLCP activity in a dose-dependent manner, thereby increasing p-MLC and increasing contraction (65). Therefore, we verified the enhancement of ROCK activity by apatinib and HSD in **mouse models of gastric cancer** by measuring p-MYPT-1 and p-MLC protein expression. Our results also revealed that apatinib treatment caused significant upregulation of p-MLC and p-MYPT-1 in mouse models of gastric cancer. These changes can lead to vascular contraction and were reversed by concomitant treatment with the non-specific ROCK inhibitor Y27632, confirming our hypothesis.

Previous studies have demonstrated that increased ET-1 secretion and disordered NOS are the key mechanisms through which TKIs induce hypertension (66, 67). *In vivo* and *in vitro* studies have shown that vatalanib, a VEGFi, decreases the activation of eNOS and NO in mice, resulting in endothelial dysfunction and vascular hypercontractility (68, 69). In the present study, apatinib treatment increased ET-1 and iNOS and decreased eNOS in the aorta. Furthermore, Y27632 significantly downregulated ET-1 and iNOS and increased eNOS expression in the aorta, indicating that the RhoA/ROCK signaling pathway could be involved in the regulation of the ET system and NOS.

The CD31 antigen, which is expressed on all continuous endothelia, is a single-chain type-1 transmembrane protein belonging to the immunoglobulin superfamily. The constitutive expression of the CD31 antigen on endothelial cells defines the morphology of these cells (70). eNOS uncoupling and the resulting oxidative stress are major drivers of endothelial dysfunction and atherogenesis (71, 72). During endothelial injury, however, a reduction in NO release may lead to vasoconstriction and arterial obliteration (70, 73). Therefore, the expression of the endothelial markers CD31 and NOS can be used to evaluate endothelial integrity (74). The integrity of the small vascular endothelium was disrupted, which may promote fibroblast activation, leading to progressive vascular fibrosis.

In the superior mesenteric artery, the effect of apatinib on the endothelial integrity of small vessels was observed by immunohistochemistry. Apatinib and the HSD impaired the endothelial integrity of small vessels. In addition, histological methods and qRT–PCR showed that apatinib and HSD resulted in vascular lumen narrowing and increased COLI and COLIII expression, indicating vascular hypertrophy and increased vascular stiffness. Therefore, apatinib and HSD promoted aortic remodeling. Y27632 can improve the integrity of the small vessel endothelium and the fibrosis of aortic vessels.

Increased hemodynamic stress imposed by hypertension on the LV leads to major alterations in the myocardium, including hypertrophy and structural remodeling (75, 76). The RhoA/ROCK pathway is associated with myocardial hypertrophy (77). In our study, echocardiography revealed that apatinib induced hypertension through the RhoA/ROCK pathway and eventually led to left ventricular hypertrophy, consistent with previous studies. Myocardial fibrosis is a common pathophysiological manifestation of hypertensive heart diseases (78). Masson staining and immunohistochemistry analysis of α-SMA were performed to detect myocardial fibrosis. In addition to α-SMA, we also examined the expression of COLI and COLIII in myocardial tissues by qRT–PCR. Furthermore, left ventricular function was measured by echocardiography. The results suggested that apatinib and the HSD led to myocardial fibrosis and left ventricular hypertrophy after the increase in BP. ROCK inhibitors may improve ventricular hypertrophy and heart function in hypertensive rats (79, 80). In this study, we found that Y27632 reversed myocardial fibrosis and left ventricular hypertrophy induced by apatinib and the HSD.

In conclusion, our experiment found that both apatinib and an HSD not only elevated blood pressure in a gastric cancer mouse model through the RhoA/ROCK signaling pathway but also promoted vascular remodeling and left ventricular hypertrophy. Y27632, which improved aortic remodeling, fibrosis, endothelial dysfunction, and superior mesenteric artery endothelial injury, as well as left ventricular dysfunction and cardiac fibrosis in mice, is a potential drug for the treatment of hypertension caused by apatinib and an HSD.

Of course, there are limitations in the study. First, a comparison of ROCK inhibitors is lacking. Fasudil, the best known ROCK inhibitor, is clinically relevant and clinically approved in Japan and China. In the current experiment, fasudil was not used in a comparative mode to Y-27632, which weakens our findings. Additionally, as ARHGEF11 is known to act through RhoA, it was expected that it is involved in the RhoA/ROCK pathway, and ARHGEF11 gene knockout may have attenuated activation of the ROCK pathway. Therefore, ARHGEF11 gene knockout mouse models should be established to further elucidate the mechanism of the RhoA/ROCK signaling pathway. Moreover, *in vitro* experiments were not performed, and future studies applying ARHGEF11 gene knockout technology will provide a more detailed view of the molecular changes, combined with cellular level changes, in hypertension induced by activating the RhoA/ROCK signaling pathway.

## Data Availability Statement

The original contributions presented in this study are included in the article/supplementary material, further inquiries can be directed to the corresponding author/s.

## Ethics Statement

The animal study was reviewed and approved by the Ethics Committee Board of Lanzhou University Second Hospital (license number: D2019-101).

## Author Contributions

WW performed the experiments and wrote the initial draft of the manuscript. JY, QW, CL, and WW designed the study. HZ, CZ, and XF analyzed the data. All authors have read and approved the final manuscript.

## Conflict of Interest

The authors declare that the research was conducted in the absence of any commercial or financial relationships that could be construed as a potential conflict of interest.

## Publisher’s Note

All claims expressed in this article are solely those of the authors and do not necessarily represent those of their affiliated organizations, or those of the publisher, the editors and the reviewers. Any product that may be evaluated in this article, or claim that may be made by its manufacturer, is not guaranteed or endorsed by the publisher.
