# Corrigendum: Research on the mechanism and prevention of hypertension caused by apatinib through the RhoA/ROCK signaling pathway in a mouse model of gastric cancer

**DOI:** 10.3389/fcvm.2022.1070597

**Published:** 2022-10-27

**Authors:** Wenjuan Wang, Caie Li, Chenchen Zhuang, Haodong Zhang, Qiongying Wang, Xin Fan, Miaomiao Qi, Runmin Sun, Jing Yu

**Affiliations:** Department of Hypertension Center, Lanzhou University Second Hospital, Lanzhou, China

**Keywords:** hypertension, apatinib, gastric cancer, RhoA/ROCK signaling pathway, Y27632

In the published article, there was an error in the author list. Author Qingjian He was erroneously included and should have been thanked in the Acknowledgements statement instead. The corrected author list and Acknowledgements appear below.

Wenjuan Wang, Caie Li, Chenchen Zhuang, Haodong Zhang, Qiongying Wang, Xin Fan, Miaomiao Qi, Runmin Sun and Jing Yu^*^

## Publisher's note

All claims expressed in this article are solely those of the authors and do not necessarily represent those of their affiliated organizations, or those of the publisher, the editors and the reviewers. Any product that may be evaluated in this article, or claim that may be made by its manufacturer, is not guaranteed or endorsed by the publisher.

